# On the Climate of Lake Superior

**Published:** 1856-02

**Authors:** Charles Whittlesey

**Affiliations:** Eagle River, Ill.


### THE NORTH-WESTERN

### MEDICAL AND SURGICAL JOURNAL.

(NEW SERIES.)

VOL. V. FEBRUARY, 1856. NO. 2.

### ORIGINAL COMMUNICATIONS.

ARTICLE I.—*On the Climate of Lake Superior.*—By CHAS. WHITTLESEY, of Eagle River, Ill.

PROF. W. B. HERRICK, CHICAGO:—

*Dear Sir:*—My brother, Dr. S. H. Whittlesey, of the Copper Falls Mine, has shown me your article on the health and longevity of the Lake Superior region. This subject has a deep interest for the southern invalid, as, also, those who desire the growth of the upper country. I became convinced, in 1845–6, from the observed effects of the climate, in summer, upon persons with diseased lungs, that it is a mistake to send them *South* in the early stage of the disease. This idea was opposed to the theory, and to the practice, of the physicians of my acquaintance, and was simply the result of observation. I have often asked those who were laboring with coughs and sweats, on the way up, how their lungs were affected by a stay of one or two months here. The answer is, generally, "I have forgotten all about my lungs." In other cases, of great exhaustion, where there appeared to be much disorganization of the breathing apparatus, and bleeding, I do not feel confident that the sufferer's life will be prolonged by a residence on this lake. In two instances, it appeared to have this effect; but, in four or five others, I thought the influence was not good. This depends somewhat upon the season of the year they arrive, and upon their mental energy.

I am happy to see the faculty discussing the question, and like the general tone and deductions of your article. Great good may be produced by a judicious recommendation to invalids, respecting a summer residence here. The time is at hand, when, at Marquette, Copper Harbor, or La Pointe, they may be comfortably situated.

Having spent a large part of ten years on the waters of the lake, I feel no hesitation in saying that, for pulmonary difficulties, and for general debility, more benefit is to be expected than in any portion of the United States, hitherto resorted to for health. I write this, however, to assist you somewhat by furnishing some facts in Meteorology, in addition to those at your command when your article was written.

As you regard Barometric pressure as one of the elements involved, I enclose the best statement that (as far as I know) can now be made, there being no readings for the Winter months —a season when the fluctuations are greatest.

The only other place, of an elevation near ours, to which I can now refer in comparison, is Rochester, N. Y.; and you will see that the extremes for the same months are greater here than there.

STATEMENT, *showing the extreme fluctuations of the Barometer on Lake Superior, at or near water level, in the Summer months, compared with Rochester, N. Y.*

| *Place and Date.* | | *Highest Reading.* | *Lowest Reading.* | *Difference.* | *Authority.* |
|---|---|---|---|---|---|
| *Copper Harbor.* | | | | | |
| July, 1847, | - | 29.710 | 28.069 | 1.641 | Prof. W. W. Mathew, Six readings a day. |
| August, " | - | 29.712 | 29.240 | 0.472 | |
| July, 1848, | - | 29.562 | 28.968 | 0.594 | Joseph S. Kendall, Readings hourly. |
| August, " | - | 29.788 | 28.736 | 1.052 | |
| Sept. " | - | 29.800 | 28.978 | 0.822 | |
| *La Pointe.* | | | | | |
| July, 1849, | - | 29.85 | 29.14 | 0.710 | Chas. Whittlesey, Readings not regular. |
| August, " | - | 29.63 | 29.18 | 0.450 | |
| Sept. " | - | 29.75 | 29.26 | 0.490 | |

| | | | | |
|---|---|---|---|---|
| *Rochester, N. Y.* | | | | |
| July, 1852, - | 29.723 | 28.947 | 0.776 | Prof. C. Heywood, Six times a day. |
| August, " - | 29.723 | 29.203 | 0.529 | |
| Sept. " - | 29.808 | 28.735 | 1.073 | |
| July, 1853, - | 29,800 | 29.323 | 0.480 | Prof. C. Dewey, Daily readings. |
| August, " - | 29.750 | 29.230 | 0.520 | |
| Sept. " - | 29.840 | 29.070 | 0.770 | |

The observations for temperature, made at Copper Harbor by the army surgeons, having been all taken at *lake level,* do not give a correct average. The thermometer is so affected that it reads higher near large bodies of water, and thus registers, kept at the same time on the highlands adjacent, are materially lower, especially in Winter. I believe I have transcripts of all the readings that have been recorded within ten years, having copied those of which I have any knowledge.

The accompanying table shows the extreme range at lake level, and the lowest depression during Winter, at several points in the interior.

Thermometers differ so much, that a few observations, with instruments not compared, may vary several degrees from the truth; but we must use such materials as we have. For the Summer months I have no observations on the highlands.

A STATEMENT, *showing the extreme fluctuations in Thermometrical Readings on Lake Superior.*

| *Places at the Level of the Lake.* | *No. of obs. per day.* | *Lowest in Winter.* | *Highest in Summer.* | *Mean of Summer.* | *Authority.* |
|---|---|---|---|---|---|
| Grand Island, 1842,...... | — | — 3 | — | | Not known. |
| Copper Harbor, 1844–5,. | 3 | 0 | 93 | 62.40 | Army Surgeons. |
| do. 1845–6,. | " | — 9 | 94 | 62,40 | do. |
| do. 1847,.... | 6 | —— | 80 | 58.40 | Pr. W.W. Mather. |
| do. 1848,.... | 12 | —— | 89 | 68.08 | S. S. Kendall. |
| Elm River, 1848,.......... | — | — 7 | — | | Not known. |
| La Pointe, 1849,.......... | — | —— | 81 | 61.15 | C. Whittlesey, |
| Ontonagon, 1853–4,...... | 3 | — 2 | | | A. Stockley. |
| do. 1854–5,...... | " | — 4 | | | H. S. Taft, M. D. |
| Eagle River, " ...... | — | —17 | | | C. Whittlesey. |
| Eagle Harbor, 1853–4.... | — | —22 | | | Not known. Single observations. |
| do. 1854–5,... | — | —26 | | | |
| *For Places in the Interior.* | | | | | |
| Portage Lake, 1853–4,.. | 3 | —30 | | | C. H. Palmer. |
| Cliff Mine, " .. | — | —36 | | | Single observation |
| Copper Falls, " .. | — | —19 | | | " " |
| Northwest Mine, 1854-5, | — | —30 | | | " " |
| Phœnix Mine, " | — | —26 | | | " " |
| Cliff Mine, " | — | —32 | | | " " |

Mean of Extreme Cold for 5 years, at lake level,............. —10
Same for the two years in the Interior,........................ —29

The best established point in the Meteorology of Lake Superior, at lake level, is the *mean* of the *Summer months, June, July, and August.* The average of five years is 65.28, which is certainly a very comfortable temperature. It is a settled fact, that a pure northern atmosphere invigorates the animal system. A southern climate may be very pleasant, where it is uniform and moderate; but does it not *enervate* the animal system, *invariably?* Those invalids who stand in fear of the chills of our northern summers, will perceive that, by the thermometer, we have a delicious mean between hot and cold; and while, for mere personal comfort, no part of North America is to be preferred in Summer, especially for depressed and declining constitutions, there is probably none better calculated, as a permanent residence, to prolong life in healthy individuals.

The Hygrometric state of the air has not been so much observed. I should say that the warm months are moist, and the cold months *very dry.* In the Spring, when the snow leaves the ground, (if there is no rain, and it is not to be expected for some weeks,) the old timber and brush is so dry as to cause raging fires in the woods at once.

The army surgeons kept a "wet bulk" register at Copper Harbor for two years, which are the only observations I know of on moisture. I give you the results, but they do not at all correspond to my ideas of the Hygrometic state of the atmosphere in Winter. My attempts to use the wet bulk, in our cold, sharp, freezing months, have been a failure; and I, therefore, suspect the results given at Fort Wilkins. They are as follows:—

| | | *Wet Bulk Mean.* | *Difference for Moisture.* |
|---|---|---|---|
| Mean of Summer, 1844, . . . | 62.42 | 56.22 | 6.20 |
| Winter mean, 1845, . . . . | 22.97 | 21.99 | 0.98 |
| Summer, . . . . . . . . . | 61.40 | 54.71 | 6.69 |
| Winter, 1846, . . . . . . . | 21.16 | 20.95 | 0.21 |

The land and sea breezes of which you speak are common to all the lakes, and all large bodies of water, whether fresh or salt. In calm weather, on Lake Erie, during the Summer months, the lake captains count upon an off-shore breeze about eight o'clock in the evening, as they do on the ocean, and this on the North as well as the South shore. Where there are highlands, these diurnal changes are more conspicuous. The cause you will readily perceive.

As to the irregular oscillations of the lake's surface, which is also common to all of our fresh water seas, (and probably to the ocean,) after some years of observation and measurement, I am nearly convinced that they are not due to sudden changes in Barometric pressure; but my investigations are not sufficiently advanced to give a theory of the agent which produces them. The general and the annual rise and fall of surface, is a sure index of the combined Meteorology over the vast tract drained into and from the great North American lakes. The statistics in my possession, I think, establish this point, about which a great amount of mystery has been thrown, by traditions that do not agree with facts. A series of wet and dry seasons produces a rise, and of cold and dry ones a fall, as in all rivers and inland bodies of water.

